# After San Juan

**Published:** 1899-02-18

**Authors:** 


### AFTER SAN JUAN.

In a recent number of the *Contemporary Review* a realistic description of "The Night After San Juan" is given by Stephen Bonsal, showing up, in lurid colours, the after-horrors of war. With all the experience of past fights, with all the advantages of modern science, and in spite of the elaborate and, on paper, apparently perfect machinery of medical and ambulance services, the fate of the brave men who fought in Cuba seems to have been in many instances little better than that of their predecessors in less civilised ages.

The taking of San Juan was an achievement dearly paid for in the loss, often the unnecessary loss, of brave and valuable lives. It seems too clear that suffering untold and many deaths must have resulted from the absence of prompt and efficient medical aid. Hundreds of men fell that day. "As we gazed upon it from afar the charge of the two gallant infantry brigades up the slopes leading to the heights where the San Juan Fort was perched resembled nothing so much as a great wave sweeping slowly, but surely, in from the sea. . . . in one place right under the fort the human wave rose and ran out into a point. You could count on the fingers of your hand the brave men who were leading it, and even as you counted they grew fewer, their arms going wildly up in the air as they fell. Then, with a weak and tired cheer, half a dozen men came out upon the open ground in front of the block-house, looking strangely tall against the sky line." And so the fort was taken, for the Spaniards had fled.

But what of the wounded? Back on the San Juan Creek the Central Emergency Hospital had been established, and to this, carried on blankets, caked with mud and wet, and so rotten that they would tear through, throwing the occupant upon the ground, those who had fallen in the battle were taken. Of proper stretchers there were none. These had been abandoned in the rush forward. But there was not much shelter even here. Alas, for our "cultured Christian age"! Several of the surgeons and attendants bravely doing their work throughout that terrible day, were picked off by sharpshooters, "to whom it seemed that the Red Cross which flew over the hospital station, and which was worn by those who were charged with the care of the wounded, was indeed a shining and attractive mark, and one rather sought after than avoided." Time pressed, and the peril was great; therefore the search for the wounded was a superficial one, and many were not discovered till the following day or even till the third day after the fight. Of others, who had strength to struggle into the shelter of the jungle, who shall say how many died there of wounds, exposure, and starvation?

Mr. Bonsal estimates that at least one-third of the men wounded on July 1st received no attention, and were not brought back to the division hospital until the afternoon of July 3rd. There were practically no ambulances. Had they been at hand, "ambulances, without which it is reasonable to suppose—at least we had supposed—no civilised Power would enter upon an aggressive war, much less upon a campaign in which we had the advantage of choosing both our own time and the field of operations, the outrageous treatment which our wounded suffered, and the barbarous scenes which we were called upon to witness upon this and the following days would have been prevented."

The whole story is one of blunder. For want of transportation the regimental surgeons were unable to take their medicine chests to the front, therefore the most elementary first aid was all that could be given to the soldiers where they fell. From constant, unchanging wear in drenching rains, the men's clothing was so rotten that it almost fell to pieces when their wounds were dressed, and the greater number arrived half or wholly naked at the hospital, where there was not one scrap of clothing or bedding to cover them. To convey these seriously injured men down to the base hospital at Siboney but three ambulances were available, and the wounded were packed into rough army waggons for the long journey. "The waggons were large and roomy, but there were no sections or compartments, nothing for the poor jostled patient to hang on to as the springless vehicle jolted over the rugged road; and the siding and flooring of the waggons were rough and splintered by the weight of the heavy barrels, the cartridge boxes, and the other heavy freight that they had carried this very day. In consequence many a wounded man was taken out covered with splinters, which had penetrated deep into his flesh. . . ." But this was not the worst. There being no sort of means by which the wounded could retain their positions upon the floor of the waggons, not even a tail-board, the result, as the waggons tilted back and forth over the roughness of the trail, plunged down the banks of streams, to be rushed at a gallop up the other side, is not one to be dwelt upon. Hear Mr. Bonsal:—

"It was at a ford a mile back from the dressing station . . . that I first realised the suffering which the absence of proper transportation entailed upon the wounded. Here it was that I came to understand that the men who I had heard crying out as we passed them on the dark trail, 'Stop, stop! For God's sake let me get out and die in the grass,' were not delirious, but in conscious agony, suffering more than the strongest man could bear. . . . Having no way of retaining their positions through all the jolting and jarring, the sliding backward and forward, the wounded whom but half an hour before I had seen each in his place, and as far as space was concerned comparatively comfortable, now lay huddled together in indescribable confusion. There they lay, a squirming, writhing mass of naked, blood-stained, and bandaged limbs. . . . It was hard to realise, as I heard their pitiful cries, that these were the same brave, patient fellows who had smiled so cheerfully as we helped them into the waggon half an hour before, with the thought that, at least for the present, their greatest sufferings were at an end. You would have been moved to indignation had the bodies that were heaped together in this way been the bodies of the dead. . . . Not a few men were taken out of the waggons dead. Many who had been placed in the waggons with the assurance from the surgeons at the front that they would be about in a few days and could return to the colours, were found delirious and shrieking with horror, and living over again the scenes of suffering which they had witnessed, and in which they had been involved in the journey down from the front."

But at last the hospital enclosure was reached, the men carried in and laid down in long files on the wet grass to wait their turn for examination and treatment, while up and down the ranks the younger surgeons hurried to look for urgent cases, in which every second is of importance. "Each in his turn, . . . . the wounded are carried up to the examination table, where white men, and black, and yellow, succeed one another like the painted slides of a stereopticon. . . . Our regulars, our own people, the blacks who have won to-day in every quarter of the extended field a fame that will never die, and the Cubans, who have done what they could do, follow one another across the operation table, showing upon their torn and mutilated bodies every conceivable wound that man can suffer. And not from a single one of them is heard a word of complaint."

The hopelessly wounded were laid beneath a great ceiba-tree, wrapped in a blanket, and with a bundle of guinea grass for a pillow. "Now and again a rough soldier . . . softened by the scenes of suffering that surround him into the most tender of nurses, picks his way carefully in and out of the rows of the dying, pouring upon their parched lips the precious drops of water," sponging their foreheads with alcohol and vinegar, and doing what may be done to alleviate their sufferings in those last moments.

What a tale of needless preventable suffering, which a little forethought, a little method, might surely have altogether avoided! There is but one satisfactory statement in Mr. Bonsal's article, and that is his testimony to the great efficacy of the simple first-aid bandages which had been distributed to the men some days before the fight. They were excellently adapted to the hasty dressing which was all that was possible on the field, and "undoubtedly saved many lives."

